# *Ex vivo* miRNome analysis in *Ptch1^+/−^* cerebellum granule cells reveals a subset of miRNAs involved in radiation-induced medulloblastoma

**DOI:** 10.18632/oncotarget.11938

**Published:** 2016-09-10

**Authors:** Barbara Tanno, Gabriele Babini, Simona Leonardi, Paola Giardullo, Ilaria De Stefano, Emanuela Pasquali, Andrea Ottolenghi, Michael J. Atkinson, Anna Saran, Mariateresa Mancuso

**Affiliations:** ^1^ Laboratory of Biomedical Technologies, Agenzia Nazionale per le Nuove Tecnologie, l'Energia e lo Sviluppo Economico Sostenibile (ENEA), Rome, Italy; ^2^ Department of Physics, University of Pavia, Pavia, Italy; ^3^ Department of Radiation Physics, Guglielmo Marconi University, Rome, Italy; ^4^ Department of Sciences, Roma Tre University, Rome, Italy; ^5^ Helmholtz Zentrum München, German Research Center for Environmental Health, Institute of Radiation Biology, Neuherberg, Germany

**Keywords:** miRNA, X-rays, medulloblastoma, Shh, GCPs

## Abstract

It has historically been accepted that incorrectly repaired DNA double strand breaks (DSBs) are the principal lesions of importance regarding mutagenesis, and long-term biological effects associated with ionizing radiation. However, radiation may also cause dysregulation of epigenetic processes that can lead to altered gene function and malignant transformation, and epigenetic alterations are important causes of miRNAs dysregulation in cancer.

*Patched1* heterozygous (*Ptch1^+/−^*) mice, characterized by aberrant activation of the Sonic hedgehog (Shh) signaling pathway, are a well-known murine model of spontaneous and radiation-induced medulloblastoma (MB), a common pediatric brain tumor originating from neural granule cell progenitors (GCPs). The high sensitivity of neonatal *Ptch1*^+/−^ mice to radiogenic MB is dependent on deregulation of the *Ptch1* gene function. *Ptch1* activates a growth and differentiation programme that is a strong candidate for regulation through the non-coding genome. Therefore we carried out miRNA next generation sequencing in *ex vivo* irradiated and control GCPs, isolated and purified from cerebella of neonatal WT and *Ptch1*^+/−^ mice. We identified a subset of miRNAs, namely let-7 family and miR-17∼92 cluster members, whose expression is altered in GCPs by radiation alone, or by synergistic interaction of radiation with Shh-deregulation. The same miRNAs were further validated in spontaneous and radiation-induced MBs from *Ptch1*^+/−^ mice, confirming persistent deregulation of these miRNAs in the pathogenesis of MB.

Our results support the hypothesis that miRNAs dysregulation is associated with radiosensitivity of GCPs and their neoplastic transformation *in vivo*.

## INTRODUCTION

MicroRNAs (miRNAs) are short segments (19–25 nucleotides) of nonprotein-coding single-stranded RNA; by binding to the 3′ UTRs of their target mRNAs, they interfere with target gene expression acting primarily at post-translational level. miRNAs control a wide variety of cellular functions such as apoptosis, cell proliferation, differentiation, metabolism, stem cell renewal and stress response. It is now well accepted that a single miRNA has the potential to mediate translation of hundreds of targets and, conversely, several miRNAs can regulate the expression of one gene [1–3]. There are several reports indicating that more than half of the miRNAs genes are located in cancer-associated genomic regions or in fragile sites, thus implying a key role of miRNAs in cancer biology through control of expression of relevant target mRNAs that facilitate tumor growth, invasion, angiogenesis, and immune evasion [3–5].

However, most miRNAs expressed in adults are tissue-specific [6] and the pattern of miRNA target gene expression is complicated, especially in the central nervous system (CNS) [7]. The cerebellar granule cells, the most abundant neurons within the entire mammalian CNS [8] originate from granular cell precursors (GCPs), whose proliferation starts during embryonic stage, reaching maximum levels postnatally. Maturation of mouse cerebellum occurs during the first 21 postnatal days (P). During this time, GCPs progressively exit the cell cycle, move into the inner regions of the external granular layer and migrate to a position beneath the Purkinje cells, where they form the cerebellum internal granule layer. [9, 10].

GCPs proliferation is mainly regulated by Sonic hedgehog (Shh) expression and activity that, thereby, determine final cerebellar size and shape [11]. GCPs neoplastic transformation causes development of medulloblastoma (MB), the most common CNS cancer in children [12].

*Patched1* heterozygous knock-out (*Ptch1*^+/−^) mice, in which deregulation of the Shh/Ptch1 pathway stimulates proliferation and inhibits differentiation of GCPs, are considered the best characterized murine model of MB. When maintained on a CD1 genetic background, these mice show ∼ 8% of MB spontaneous rate [13, 14]. They are also characterized by radiation hypersensitivity. When exposed to ionizing radiation, as newborns, *Ptch1*^+/−^ mice display greatly accelerated tumor development suggesting that, along with its patterning role, Shh deregulation is also involved in the response to damage. Earlier studies from our laboratory showed a clear dose-dependent increase of MB incidence in mice irradiated at postnatal day 2 (P2), with doses ranging from 0.5 to 3Gy of X-rays [15, 16].

The goal of this study was to understand whether miRNome alterations play a role in radiation-induced tumorigenesis occurring in the brain of *Ptch1*^+/−^ mice after a single dose of X-rays. To this aim, we analyzed miRNA expression in *ex vivo* radiation-treated GCPs isolated and purified from cerebella of P2/P3 WT and *Ptch1*^+/−^ mice. A subset of miRNAs controlling different biological functions, whose expression was altered in primary cultures of GCPs by radiation alone or in combination with Shh-deregulation, was identified. Their persistent deregulation in radio-induced MB compared with spontaneous tumors add new insights on the radiosensitivity of GCPs and their neoplastic transformation *in vivo*.

## RESULTS

### Differentially expressed miRNAs in unirradiated and irradiated WT and *Ptch1*^+/−^ GCPs

**Figure 1:**
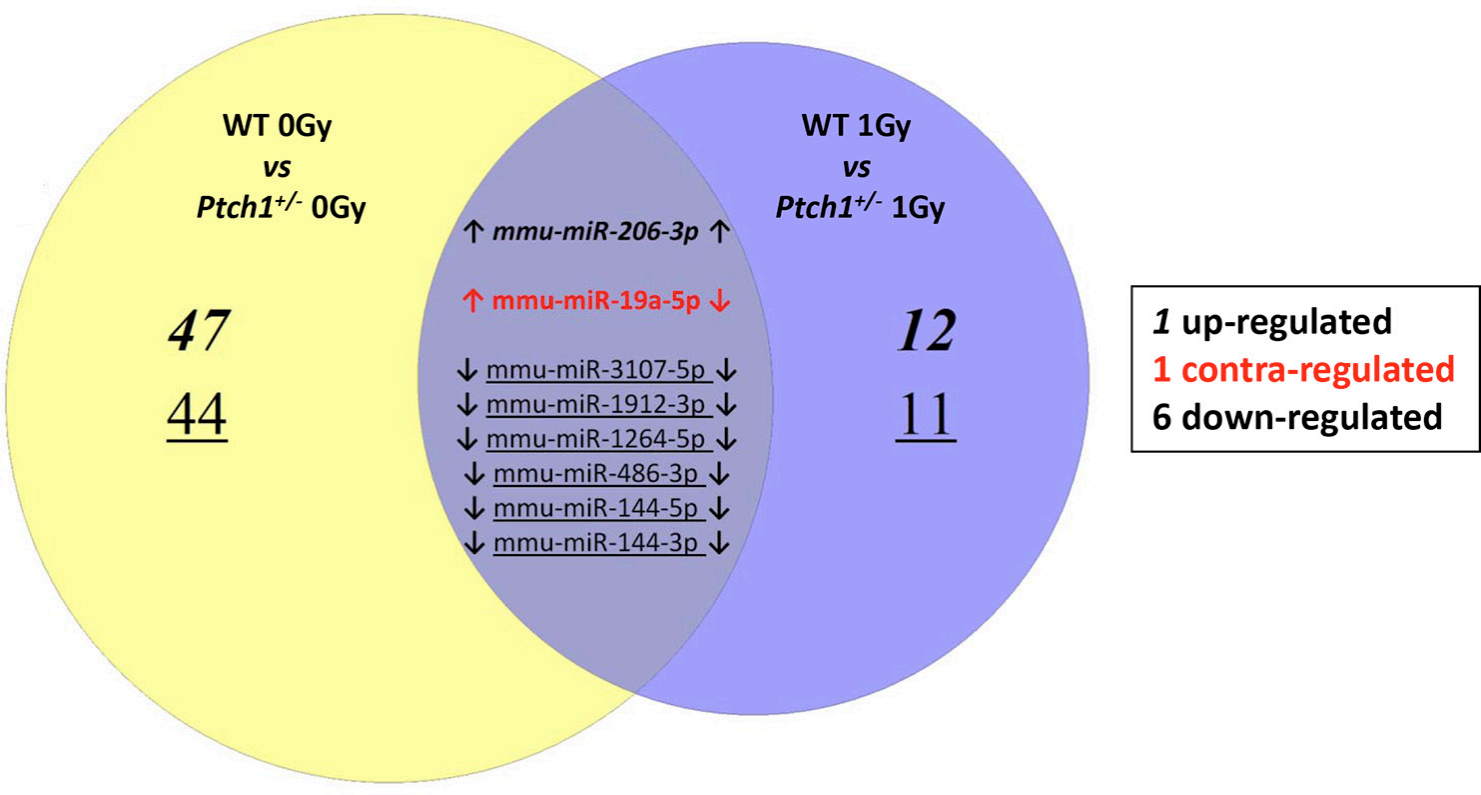
Venn Diagram describes the overlap between the statistically significant miRNAs perturbed in the unirradiated (left) and 1 Gy irradiated (right) *Ptch1*^+/−^ GCPs when compared to the corresponding WT GCPs The common miRNAs have been explicitly subclassified into both up-regulated (bold), down-regulated (underlined) or contra-regulated (red).

We first compared the baseline levels of miRNAs expressed in WT and *Ptch1*^+/−^ unirradiated GCPs, obtaining in total 99 differentially expressed miRNAs with *P* < 0.05. Of these, 49 were upregulated and 50 downregulated (Table 1). In order to better understand the role of radiation in the modulation of miRNAs dependent on Shh deregulation we compared irradiated WT and *Ptch1*^+/−^ GCPs. Results reported in Table 2 show a reduced number of statistically significant deregulated miRNAs, i.e. 31, when compared to the unirradiated condition, i.e. 99 miRNAs. Of these 31 miRNAs, 13 were up-regulated while the remaining 18 were down-regulated. By comparing the baseline levels of miRNAs expressed in WT and *Ptch1*^+/−^ GCPs with that obtained after irradiation of both genotypes, 8 common miRNAs were statistically significant (Figure 1). Only one miRNA was up-regulated (mmu-miR-206-3p), while 6 out of 8 were down-regulated (i.e., mmu-miR-3107-5p, mmu-miR-1912-3p, mmu-miR-1264-5p, mmu-miR-486-3p, mmu-miR-144-5p and mmu-miR-144-3p). One miRNA was contra-regulated (mmu-miR-19a-5p).

**Table 1:** Differentially expressed miRNAs in unirradiated WT and *Ptch1* ^+/−^ GCPs

**Down Regulated**	**miRNA**	**logFC**	**logCPM**	**LR**	***P* Value**	**FDR**
	mmu-miR-3083-5p	−4.1974	0.8867	9.3818	0.0022	0.2064
	mmu-miR-1900	−3.7672	0.6242	7.1537	0.0075	0.3005
	mmu-miR-203-5p	−3.4093	0.6784	5.6807	0.0172	0.3825
	mmu-miR-292-3p	−3.3020	1.0060	8.5166	0.0035	0.2184
	mmu-miR-10a-5p	−3.0421	1.4827	8.9714	0.0027	0.2064
	mmu-miR-5623-3p	−2.9155	0.5112	3.9011	0.0483	0.4721
	mmu-miR-410-5p	−2.7524	0.8271	4.0432	0.0444	0.4717
	mmu-miR-302d-3p	−2.7381	1.4311	4.7791	0.0288	0.4351
	mmu-miR-7071-5p	−2.7233	0.7251	5.1286	0.0235	0.4296
	mmu-miR-3105-3p	−2.5135	1.5964	7.7841	0.0053	0.2674
	mmu-miR-486-3p	−2.4254	2.2862	7.3432	0.0067	0.2868
	mmu-miR-99a-3p	−2.2438	1.2057	5.1626	0.0231	0.4296
	mmu-miR-466n-5p	−2.2344	1.2083	4.2171	0.0400	0.4520
	mmu-miR-6932-3p	−2.1863	1.7642	10.8027	0.0010	0.2064
	mmu-miR-144-5p	−2.1737	3.7198	12.0759	0.0005	0.2064
	mmu-miR-6901-5p	−1.9843	1.3443	5.0136	0.0251	0.4329
	mmu-miR-378b	−1.9694	2.4781	11.6548	0.0006	0.2064
	mmu-miR-764-3p	−1.8894	3.2648	10.8063	0.0010	0.2064
	mmu-miR-451a	−1.8064	8.7796	6.5568	0.0104	0.3385
	mmu-miR-144-3p	−1.7875	5.7266	10.6281	0.0011	0.2064
	mmu-miR-146a-5p	−1.7767	5.3909	6.0973	0.0135	0.3496
	mmu-miR-674-5p	−1.6396	2.8318	6.9645	0.0083	0.3083
	mmu-miR-670-3p	−1.5797	2.3180	4.5397	0.0331	0.4351
	mmu-miR-143-5p	−1.5461	2.5800	4.5715	0.0325	0.4351
	mmu-miR-3107-5p	−1.4811	8.5419	4.0045	0.0454	0.4717
	mmu-miR-299a-5p	−1.4605	3.4344	6.2683	0.0123	0.3385
	mmu-miR-1a-3p+1	−1.4455	5.0699	5.0182	0.0251	0.4329
	mmu-miR-1912-3p	−1.4101	5.3947	9.2711	0.0023	0.2064
	mmu-miR-145a-5p	−1.3952	4.5722	5.7745	0.0163	0.3821
	mmu-miR-34c-5p	−1.3484	12.6002	9.6274	0.0019	0.2064
	mmu-miR-1298-3p	−1.3390	7.7182	5.3479	0.0207	0.4000
	mmu-miR-1912-5p	−1.3312	6.4732	5.6105	0.0179	0.3825
	mmu-miR-448-3p	−1.2906	10.3985	8.9445	0.0028	0.2064
	mmu-miR-34c-3p	−1.2855	6.7574	6.0727	0.0137	0.3496
	mmu-miR-34b-5p	−1.2364	6.9571	4.4573	0.0348	0.4351
	mmu-miR-378c	−1.1864	5.1922	7.0133	0.0081	0.3083
	mmu-miR-205-5p	−1.1504	3.9070	4.6918	0.0303	0.4351
	mmu-miR-127-5p	−1.1287	3.0536	4.6117	0.0318	0.4351
	mmu-miR-143-3p	−1.1136	10.1214	4.0685	0.0437	0.4717
	mmu-miR-1298-5p	−1.1059	15.1169	4.7593	0.0291	0.4351
	mmu-miR-1264-5p	−1.0847	7.2065	4.9582	0.0260	0.4351
	mmu-miR-23a-3p	−1.0641	8.5010	6.2801	0.0122	0.3385
	mmu-miR-100-5p	−1.0491	6.8736	3.9090	0.0480	0.4721
	mmu-miR-411-5p	−1.0288	5.5994	5.5097	0.0189	0.3881
	mmu-miR-199a-5p+1	−0.9942	4.9267	3.8930	0.0485	0.4721
	mmu-miR-193b-3p	−0.9685	4.6473	3.9639	0.0465	0.4717
	mmu-miR-449a-5p	−0.9452	10.2619	4.8124	0.0283	0.4351
	mmu-miR-99a-5p	−0.9288	10.5266	4.6332	0.0314	0.4351
	mmu-miR-136-3p	−0.8555	7.6017	4.2042	0.0403	0.4520
	mmu-miR-342-3p	−0.8506	4.9562	3.9406	0.0471	0.4721
**Up Regulated**	**miRNA**	**logFC**	**logCPM**	**LR**	***P* Value**	**FDR**
	mmu-miR-6897-3p	4.5405	1.0441	8.4627	0.0036	0.2184
	mmu-miR-7059-5p	4.0147	0.9928	4.7370	0.0295	0.4351
	mmu-miR-28c	3.6585	0.8174	4.3903	0.0361	0.4365
	mmu-miR-466l-3p	3.5689	0.9367	4.2946	0.0382	0.4441
	mmu-miR-19a-5p	3.5647	0.9746	4.1196	0.0424	0.4686
	mmu-miR-6968-3p	3.5563	0.8124	4.4777	0.0343	0.4351
	mmu-miR-7091-5p	3.1637	1.2545	4.7972	0.0285	0.4351
	mmu-miR-8094	2.5359	2.8776	8.0084	0.0047	0.2494
	mmu-miR-6975-3p	2.5219	1.9988	5.6164	0.0178	0.3825
	mmu-miR-7026-3p	2.4464	1.1267	4.6223	0.0316	0.4351
	mmu-miR-6922-3p	2.3149	1.4225	4.0008	0.0455	0.4717
	mmu-miR-341-5p	2.2574	2.0405	6.3778	0.0116	0.3385
	mmu-miR-6948-3p	2.1380	1.7761	5.4426	0.0197	0.3947
	mmu-miR-7655-3p	2.1365	1.7140	4.4601	0.0347	0.4351
	mmu-miR-7054-5p	1.9636	1.8966	4.6641	0.0308	0.4351
	mmu-miR-1964-3p	1.9090	7.8485	9.1493	0.0025	0.2064
	mmu-miR-1249-5p	1.9018	1.5979	3.9940	0.0457	0.4717
	mmu-miR-7687-5p	1.8196	2.3073	5.1224	0.0236	0.4296
	mmu-miR-3474	1.7123	3.7113	6.8285	0.0090	0.3203
	mmu-miR-7685-5p	1.6785	2.9265	6.3411	0.0118	0.3385
	mmu-miR-101b-5p	1.6631	2.1803	4.2427	0.0394	0.4520
	mmu-miR-674-3p	1.6497	9.9090	9.4070	0.0022	0.2064
	mmu-miR-151-5p	1.6442	8.8409	9.5659	0.0020	0.2064
	mmu-miR-6900-3p	1.6415	2.9844	8.5277	0.0035	0.2184
	mmu-miR-101a-5p	1.6287	2.9754	6.5177	0.0107	0.3385
	mmu-let-7a-2-3p	1.5131	2.3205	4.3444	0.0371	0.4365
	mmu-miR-505-5p	1.5119	4.3664	7.3135	0.0068	0.2868
	mmu-miR-6988-3p	1.4400	3.4336	5.8376	0.0157	0.3781
	mmu-miR-671-3p	1.3912	6.6548	6.3138	0.0120	0.3385
	mmu-miR-130b-5p	1.3726	9.3985	8.2805	0.0040	0.2272
	mmu-miR-6538	1.3334	5.5248	4.5505	0.0329	0.4351
	mmu-miR-6960-5p	1.3164	2.8508	5.5089	0.0189	0.3881
	mmu-miR-877-3p	1.3083	7.5154	5.7332	0.0166	0.3821
	mmu-miR-128-3p+1	1.3027	14.3229	7.3407	0.0067	0.2868
	mmu-miR-342-5p	1.2992	7.1620	6.7405	0.0094	0.3245
	mmu-miR-206-3p	1.2167	5.0119	5.8813	0.0153	0.3781
	mmu-miR-672-5p	1.2130	9.9835	7.3173	0.0068	0.2868
	mmu-miR-543-3p	1.1815	7.6723	5.3757	0.0204	0.4000
	mmu-miR-485-3p	1.1733	6.1384	4.6001	0.0320	0.4351
	mmu-miR-1249-3p	1.1529	4.7582	4.7865	0.0287	0.4351
	mmu-miR-7211-5p	1.0794	3.6140	4.3603	0.0368	0.4365
	mmu-miR-301b-5p	1.0770	5.0505	4.5960	0.0320	0.4351
	mmu-let-7d-3p	1.0416	10.9884	6.0659	0.0138	0.3496
	hsa-put-28	1.0337	5.0732	4.3735	0.0365	0.4365
	mmu-miR-1306-3p	0.9907	5.9592	4.1043	0.0428	0.4686
	mmu-miR-7224-3p	0.9170	5.0916	4.4345	0.0352	0.4353
	mmu-miR-149-5p	0.9140	10.4109	4.4588	0.0347	0.4351
	mmu-miR-340-3p	0.9129	10.0818	5.0136	0.0251	0.4329
	mmu-miR-328-3p	0.8067	10.6399	3.9694	0.0463	0.4717

**Figure 2:**
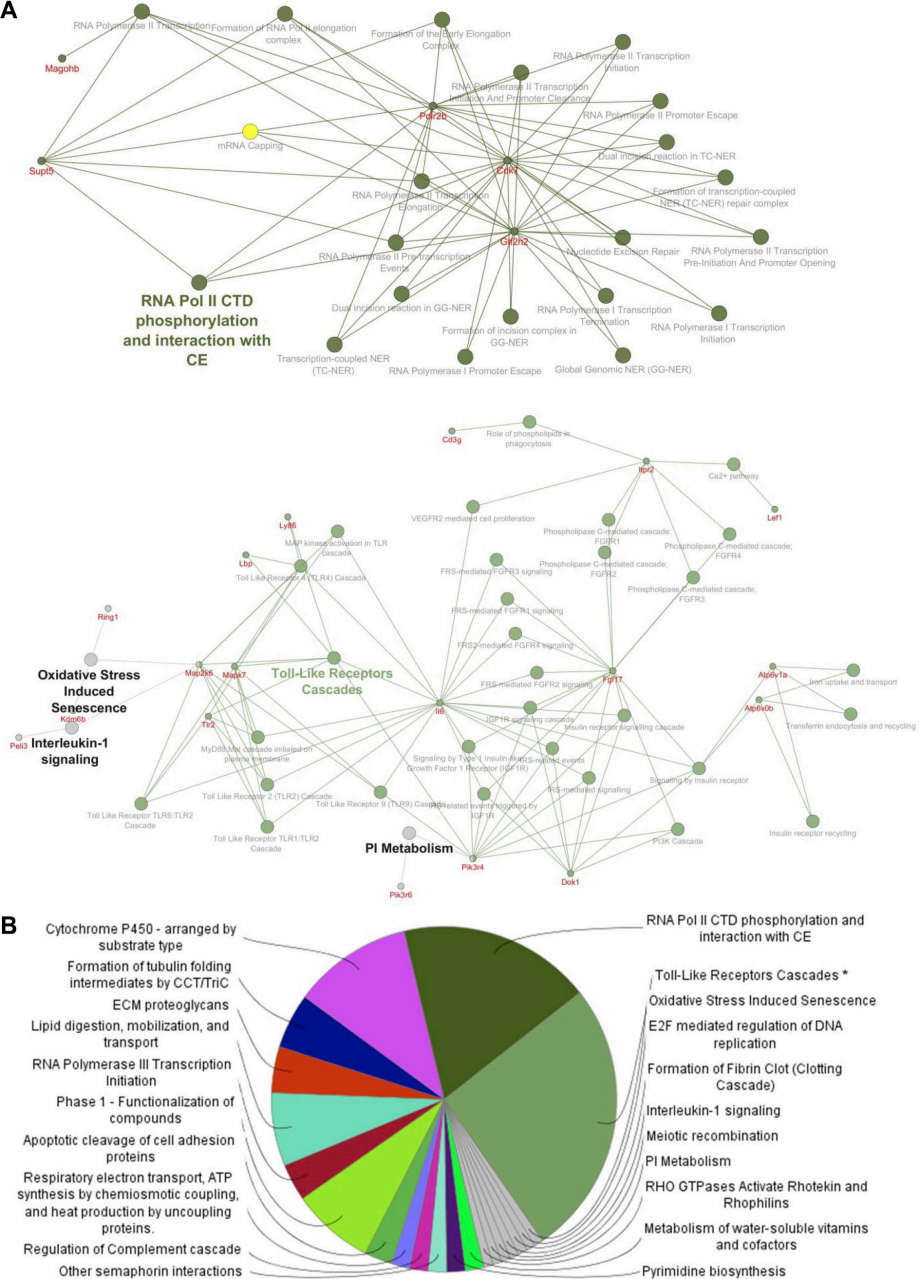
Pathway enrichment analysis was performed on the statistically significant miRNAs altered in unirradiated *Ptch1* ^+/−^ GCPs versus WT GCPs (Listed in Table 1) (**A**) Focus on some of the predicted target genes, and corresponding pathways, related to the deregulated miRNAs. (**B**) Pie chart describing the most significant Reactome pathways associated to the miRNA list.

**Figure 3:**
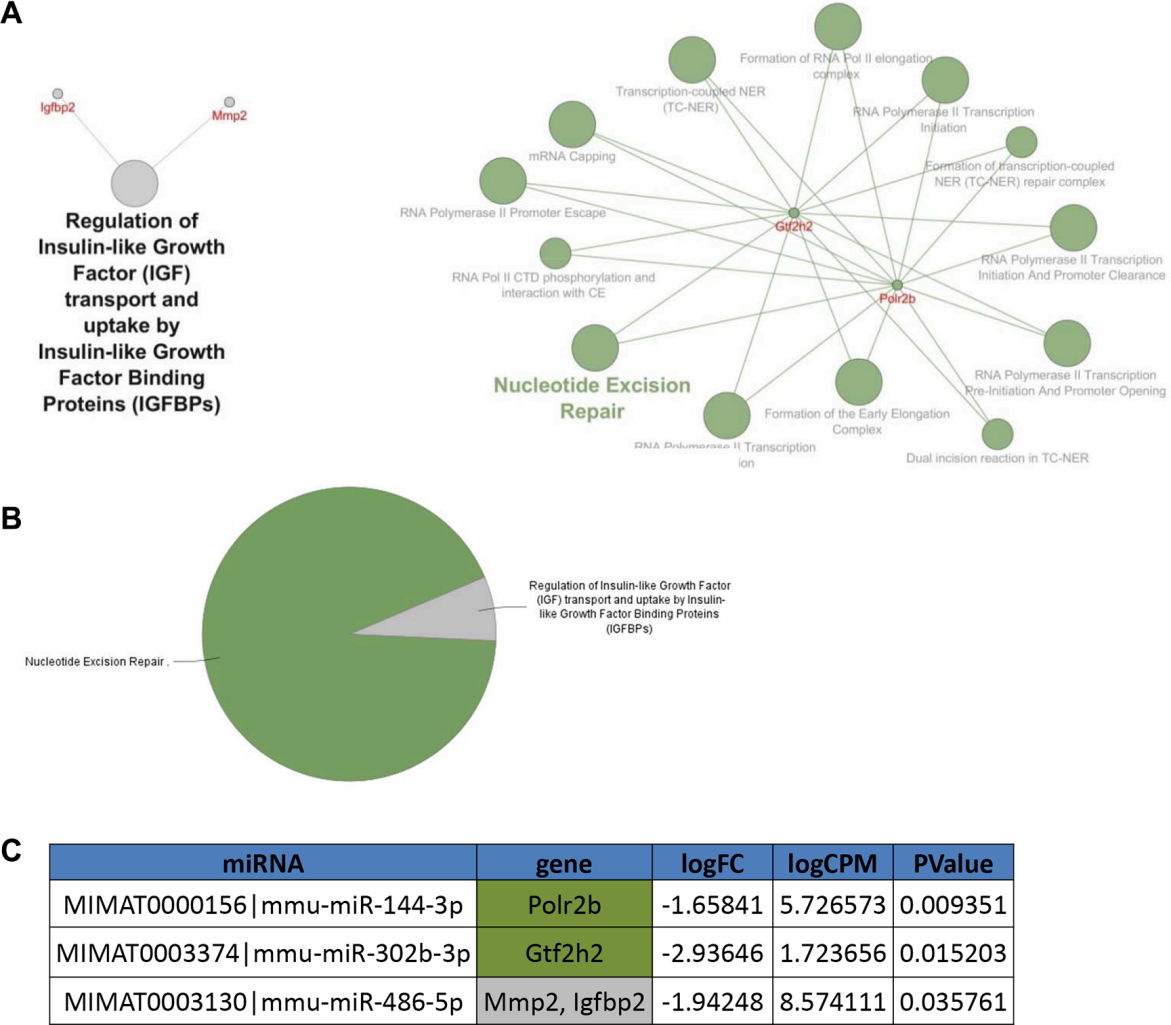
Pathway enrichment analysis was performed on the statistically significant miRNAs altered 4 hours after 1 Gy of X-rays in *Ptch1*^+/−^ GCPs versus WT GCPs (listed in Table 2) (**A**) mRNAs and corresponding Reactome pathways predicted to be altered by the deregulated miRNAs. (**B**) Pie chart showing the main pathways associated to the mRNA network. (**C**) The table highlights the miRNAs predicted to target the genes shown in the network in panel A, alongside with their log2 Fold Change, log2 Count Per Million and *p-value*.

Predicted target genes were obtained using the miRNA enrichment function of Cytoscape plugin CluePedia, selecting the top 20 genes with a miRanda SCORE > 0.6. The REACTOME database was then applied to perform a pathway analysis of the enriched gene/miRNA network. Pathway analysis revealed that, among several deregulated molecular pathways, the most significant functions affected by miRNAs deregulation in unirradiated conditions converge on RNA polymerase II transcription machinery and Toll-like receptor cascades (Figure 2A and 2B; Supplementary Figure S1), both involved in the regulation of cell cycle and survival [17, 18]. As shown in Figure 3, miRNA enrichment and pathway analysis after irradiation, highlighted only a limited number of altered functions, the most significant of which were related to Nucleotide Excision Repair (NER; *Gtf2h2* and *Polr2b* genes which are predicted targets of mmu-miR-302b-3p and mmu-miR-144-3p, respectively) and the regulation of Insulin-like Growth Factor (IGF) transport and uptake by Insulin-like Growth Factor Binding Proteins (IGFBPs) (*Mmp2* and *Igfbp2* genes are both predicted target genes of mmu-miR-486-5p).

### Misregulation of DNA repair genes in *Ptch1*^+/−^ GCPs

**Figure 4:**
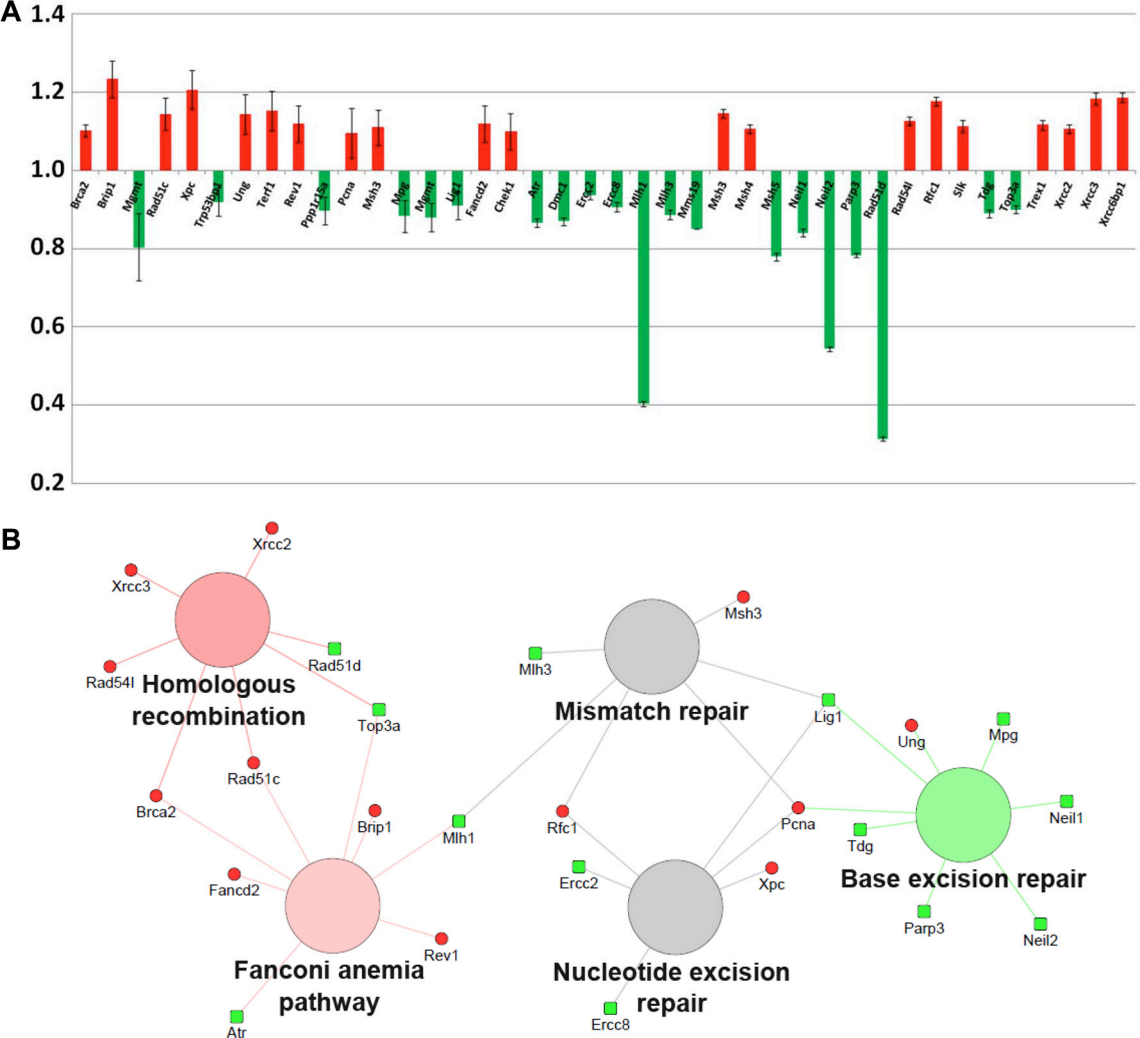
Analysis of statistically significant (*P* < 0.05) mRNAs involved in the DNA damage response in the *Ptch1* ^+/−^ GCPs after 1 Gy of X-rays with respect to the irradiated WT counterpart (**A**) Histogram of all significant genes and their corresponding fold change (error bars represent the standard deviations). (**B**) Network showing the pathway enrichment analysis of up-regulated (red) and down-regulated (green) genes. Colors of the pathway nodes reflect the number of associated genes up- or down-regulated: green if mostly down-regulated, red if mostly up-regulated and gray if equally divided.

To further investigate the perturbations induced in *Ptch1*^+/−^ GCPs after 1 Gy of X-rays *versus* the irradiated WT counterpart, the differential expression of mRNAs involved in DNA damage repair pathways was measured with the DNA Repair PCR Array and Mouse DNA Damage Signaling Pathway. Among the 84 mRNAs spotted on the arrays, data analysis highlighted that only 40 of them were significantly deregulated, of which 20 down- and 20 up-regulated, with a ratio between expression in *Ptch1*^+/−^ and WT GCPs oscillating between 1.23 and 0.31 (see Figure 4A). In particular *Brip1* gene, acting in the Fanconi anemia pathway, and *Xpc*, belonging to the NER pathway, were found up-regulated in *Ptch1*^+/−^ GCPs (1.23-fold and 1.2-fold compared to WT, respectively) while 5 mRNAs were found down-regulated more than 20%, i.e., *Rad51d* (0.31; part of the DSB repair pathway), *Mlh1* and *Msh5* (0.40 and 0.78, respectively; related to the Mismatch Repair pathway), and *Neil2* and *Parp3* (0.54 and 0.78, respectively; related to the Base Excision Repair pathway).

The complete list of statistically significant mRNAs was further analysed with the Cytoscape plugin Cluego to explore the most gene-enriched pathways. The KEGG pathways identified are shown in Figure 4B. The pathways with more up-regulated genes are represented in red (i.e., Homologous Recombination and Fanconi Anemia Pathway), while, Base Excision Repair related-genes are mainly down-regulated (in green). In grey, the pathways showing the same number of up- and down- regulated associated genes, i.e. Mismatch and NER pathways, are illustrated. Notably, network analysis confirms the altered regulation of the NER pathway seen from the NGS miRNome analysis and sheds further light on the different mechanisms of DNA damage repair in irradiated *Ptch1*^+/−^ compared to irradiated WT GCPs.

### miRNAs potentially involved in brain cancer development

**Figure 5:**
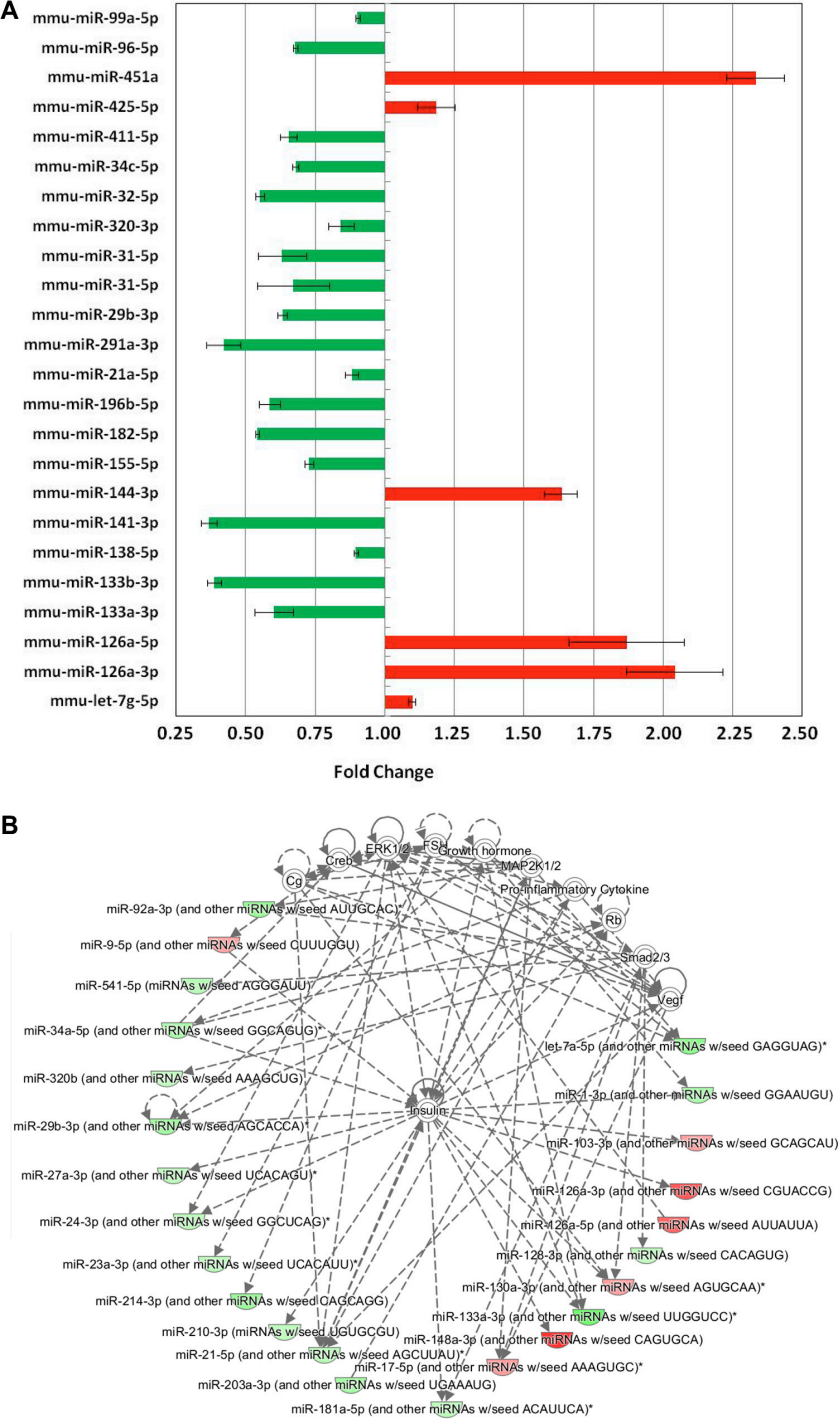
Analysis of statistically significant (*P* < 0.05) miRNAs involved in brain cancer development in *Ptch1* ^+/−^ GCPs after 1 Gy of X-rays with respect to the irradiated WT counterpart (**A**) Histogram of all significant miRNAs and their corresponding fold change (error bars represent the standard deviations). (**B**) Most significant network obtained from pathway analysis of significantly differentially expressed miRNAs in IPA shows the central role of Insulin. Color gradients reflects the corresponding miRNAs fold changes: green if down-regulated, red if up-regulated.

We asked whether some differentially expressed miRNAs identified through NGS after irradiation of WT and *Ptch1*^+/−^ GCPs matched with miRNAs whose role in brain cancer development is already well established. Using the Brain Cancer and miFinder miRNA PCR Arrays, we found 24 miRNAs showing significantly differential expression (*P* < 0.05). Among these, 18 miRNAs were down-regulated and 6 miRNAs were up-regulated (Figure 5A). Comparison of these results with miRNAs listed in Table 1, shows that 4 miRNAs, such as members of let-7 family, miR-99a, miR-34c and miR-144 were in common, while only miR-144 is in common with miRNAs listed in Table 2, indicating its potential role in MB development after irradiation.

**Table 2:** Differentially expressed miRNAs in irradiated WT and *Ptch1*^+/−^ GCPs

**Down Regulated**	**miRNA**	**logFC**	**logCPM**	**LR**	***P* Value**	**FDR**
	mmu-miR-5130	−4.6235	1.4007	5.6366	0.0176	0.9991
	mmu-miR-19a-5p	−4.3987	0.9746	4.0698	0.0437	0.9991
	mmu-miR-190b-3p	−4.3978	0.8699	4.0298	0.0447	0.9991
	mmu-miR-3072-5p	−4.2454	0.8207	4.1686	0.0412	0.9991
	mmu-miR-669l-3p	−4.2082	1.0298	3.9256	0.0476	0.9991
	mmu-miR-6943-3p	−4.1895	0.9532	4.3981	0.0360	0.9991
	mmu-miR-34a-3p	−3.4542	1.5408	7.1036	0.0077	0.9991
	mmu-miR-302b-3p	−2.9365	1.7237	5.8927	0.0152	0.9991
	mmu-miR-3061-5p	−2.8422	1.7129	4.6184	0.0316	0.9991
	mmu-miR-365-1-5p	−2.7422	1.4452	4.0480	0.0442	0.9991
	mmu-miR-486-3p	−2.2384	2.2862	3.8574	0.0495	0.9991
	mmu-miR-188-3p	−2.0717	2.0966	3.8688	0.0492	0.9991
	mmu-miR-486-5p	−1.9425	8.5741	4.4085	0.0358	0.9991
	mmu-miR-3107-5p	−1.7770	8.5419	4.2039	0.0403	0.9991
	mmu-miR-144-3p	−1.6584	5.7266	6.7546	0.0094	0.9991
	mmu-miR-144-5p	−1.4983	3.7198	4.4906	0.0341	0.9991
	mmu-miR-1912-3p	−1.4460	5.3947	6.9853	0.0082	0.9991
	mmu-miR-1264-5p	−1.1089	7.2065	3.9007	0.0483	0.9991
**Up Regulated**	**mmu-miR-6998-3p**	**4.9180**	**0.9185**	**6.9446**	**0.0084**	**0.9991**
	mmu-miR-467c-3p	4.5241	0.9938	6.7048	0.0096	0.9991
	mmu-miR-7060-3p	4.4765	1.0468	5.8776	0.0153	0.9991
	mmu-miR-467b-3p	4.1751	0.7782	4.8384	0.0278	0.9991
	mmu-miR-6918-5p	4.1393	1.0574	5.1959	0.0226	0.9991
	mmu-miR-7084-5p	4.1382	1.1594	5.1155	0.0237	0.9991
	mmu-miR-6976-3p	4.1358	0.7726	4.9216	0.0265	0.9991
	mmu-miR-7661-3p	2.3361	2.1860	4.5714	0.0325	0.9991
	mmu-miR-146b-3p	1.9034	1.7269	4.3527	0.0370	0.9991
	mmu-miR-873a-5p	1.6353	2.1819	3.8642	0.0493	0.9991
	mmu-miR-6952-3p	1.4664	3.0166	4.5177	0.0335	0.9991
	mmu-miR-672-3p	1.3691	3.8578	5.5915	0.0180	0.9991
	mmu-miR-206-3p	1.1713	5.0119	4.2435	0.0394	0.9991

Notably, evaluation of miRNAs (with a cutoff on the fold change of 1.1) and related predicted target genes in IPA highlighted a top network composed of 24 molecules which have the Insulin molecules as nodes with the highest connectivity (Figure 5B).

### Biological functions altered after irradiation

**Figure 6:**
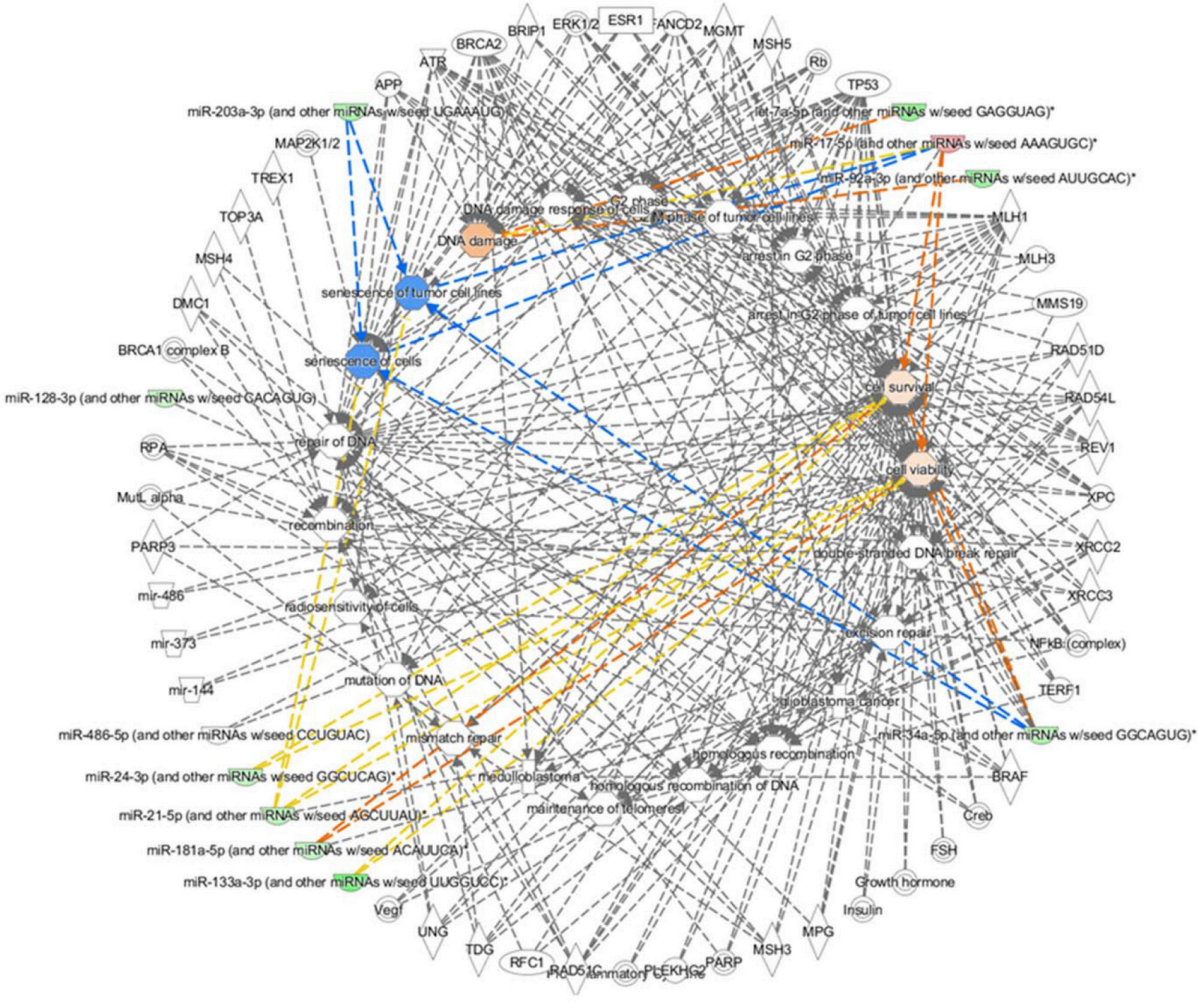
Network obtained through the Disease & Function IPA tool applied to all miRNAs and mRNAs datasets Pathways and functions are shown in the inner circle while associated miRNAs or mRNAs in the outer one. Color gradient reflects the predicted strength of activation (orange) or inhibition (blue).

The Disease & Functions IPA tool was queried to better understand the causality between the different altered functions, related to the increased incidence of MB in *Ptch1*^+/−^ mice after irradiation, and the regulatory mechanisms underpinning miRNAs and mRNAs expression changes, which were observed in the three previously described datasets. This hypothesis-driven analysis produced a network depicted as two concentric circles (Figure 6), in which the altered functions occupy the inner circle, while in the outer circle the key connected molecules (genes and miRNAs) are shown. In particular, IPA was queried for molecules known to be involved in the biological functions of cell senescence, proliferation and the different DNA damage repair pathways. The Molecule Activity Predictor (MAP), based on significantly deregulated miRNAs, suggests the inhibition of senescence (blue) and a concurrent increase of cell survival and viability (light orange) and DNA damage (dark orange), mainly due to the miRNAs let-7a, mir-17, mir-21, mir-34a, mir-92, mir-133a, mir-181a and mir-486 (Figure 6).

### miRNAs expression in unirradiated and irradiated WT and *Ptch1*^+/−^ GCPs

Among all differentially expressed miRNAs identified using NGS analysis and arrays, we performed qPCRs of those involved in the mainly perturbed biological functions (i.e., cell senescence, proliferation, regulation of Insulin-like Growth Factor [IGF] transport, and different DNA damage repair pathways).

**Figure 7:**
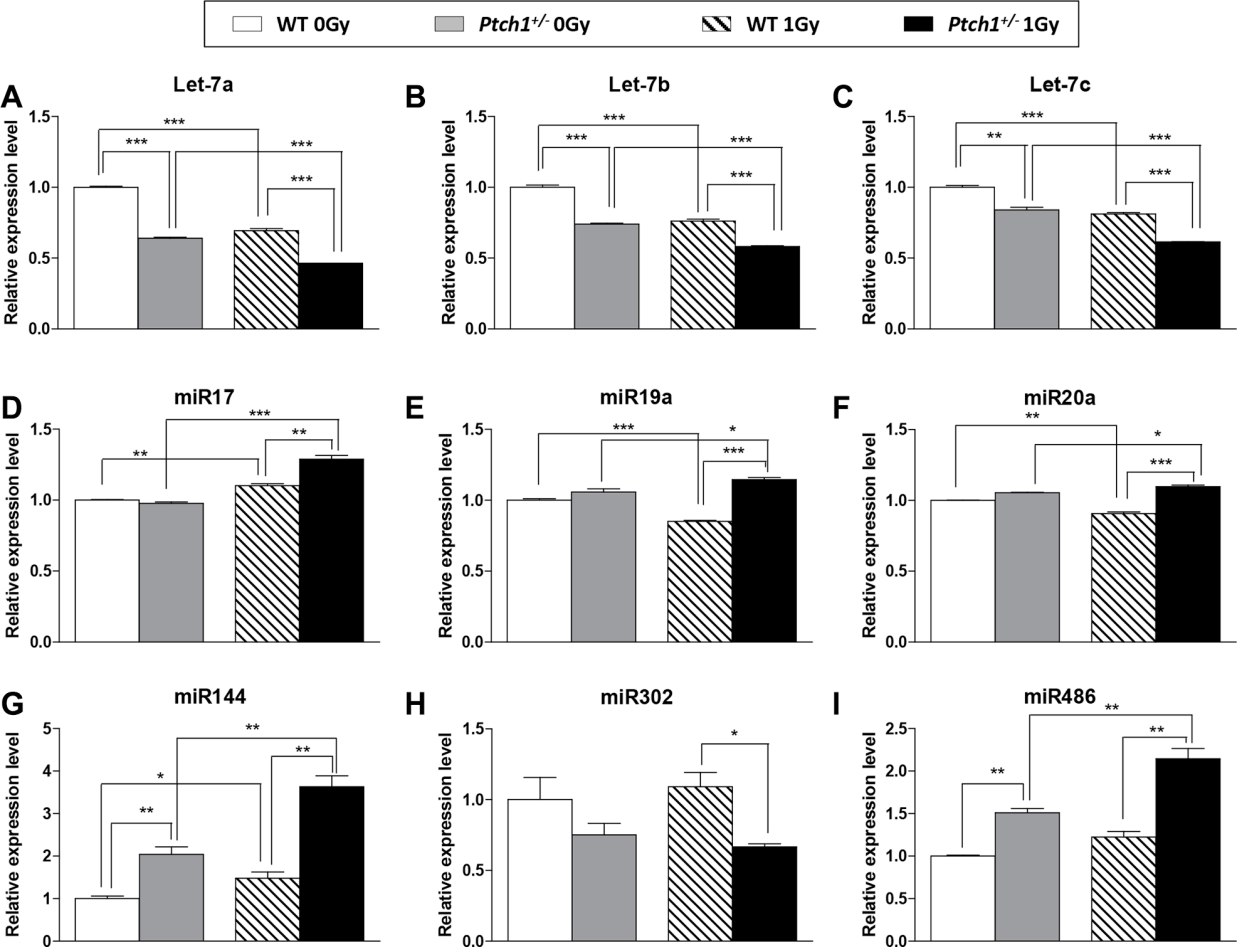
Real time PCR validation on GCPs of selected statistically significant miRNAs from NGS and arrays Each data represent the mean and the standard deviation of three biological replicates with respect to the unirradiated WT GCPs. Student's *t-test* has been performed to calculate the statistical significance of comparisons. *P ≤ 0.05; **P ≤ 0.005; ***P ≤ 0.0001.

First, we analyzed the expression of 3 members of the let-7 family, i.e. let-7a, let-7b and let-7c, involved in DNA damage response. In unirradiated condition, we found a statistically significant decreased expression of all let-7 family members in *Ptch1*^+/−^ compared with WT GCPs. Four hours after irradiation, there was a general and significant decrease in expression of all three miRNAs compared with unirradiated samples, irrespective of genotype, suggesting that not only these miRNAs are controlled by Shh deregulation, but that radiation also contributes to inhibit their expression (Figure 7A–7C). This was confirmed by the two-way ANOVA test, showing a statistically significant interaction between dose and genotype for let-7a (*P* < 0.0001) and let-7b (*P* = 0.0074) while it is not significant for let-7c (*P* = 0.15). Bonferroni post-hoc tests confirmed for all let-7 miRNAs a statistically significant difference among the two genotypes at both doses (*P* < 0.001).

Second, we analyzed 3 members of the 17∼92 cluster, i.e., miR-17, miR-19a, miR-20a, involved in cell survival and viability. Two-way ANOVA showed a statistically significant interaction between dose and genotype in all three miRNAs (*P* < 0.001). Bonferroni post-hoc tests, for unirradiated conditions, did not show any significant difference between WT and mutant cells in miR-17 and miR-19a, while a mild significance is observable in miR-20a (*P* < 0.01). On the contrary, after 1 Gy irradiation, *Ptch1*^+/−^ GCPs showed an extremely statistically significant (*P* < 0.001) higher expression levels of 17∼92 members with respect to WT cells. These results suggest that Shh deregulation and irradiation might synergize to induce a more proliferative GCPs status (Figure 7D–7F).

Finally, as shown in Figure 7G and 7H, we evaluated expression levels of miR-144 and mir-302b, both controlling NER machinery (Figure 5), showing for both miRNAs a clear dependence on *Ptch1* haploinsufficiency exacerbated by combination with irradiation, although with a reverse pattern. Similarly, for the last miRNA analyzed, i.e. miR-486 (Figure 7I), controlling the IGF signaling pathway (Figure 5), the fold change in expression was increased by irradiation, highlighting that a combination of biological functions, influenced by different miRNAs, may modify the short-term response to radiation in combination with deregulation of Shh signaling pathway.

### miRNAs expression in spontaneous and radio-induced MB

**Figure 8:**
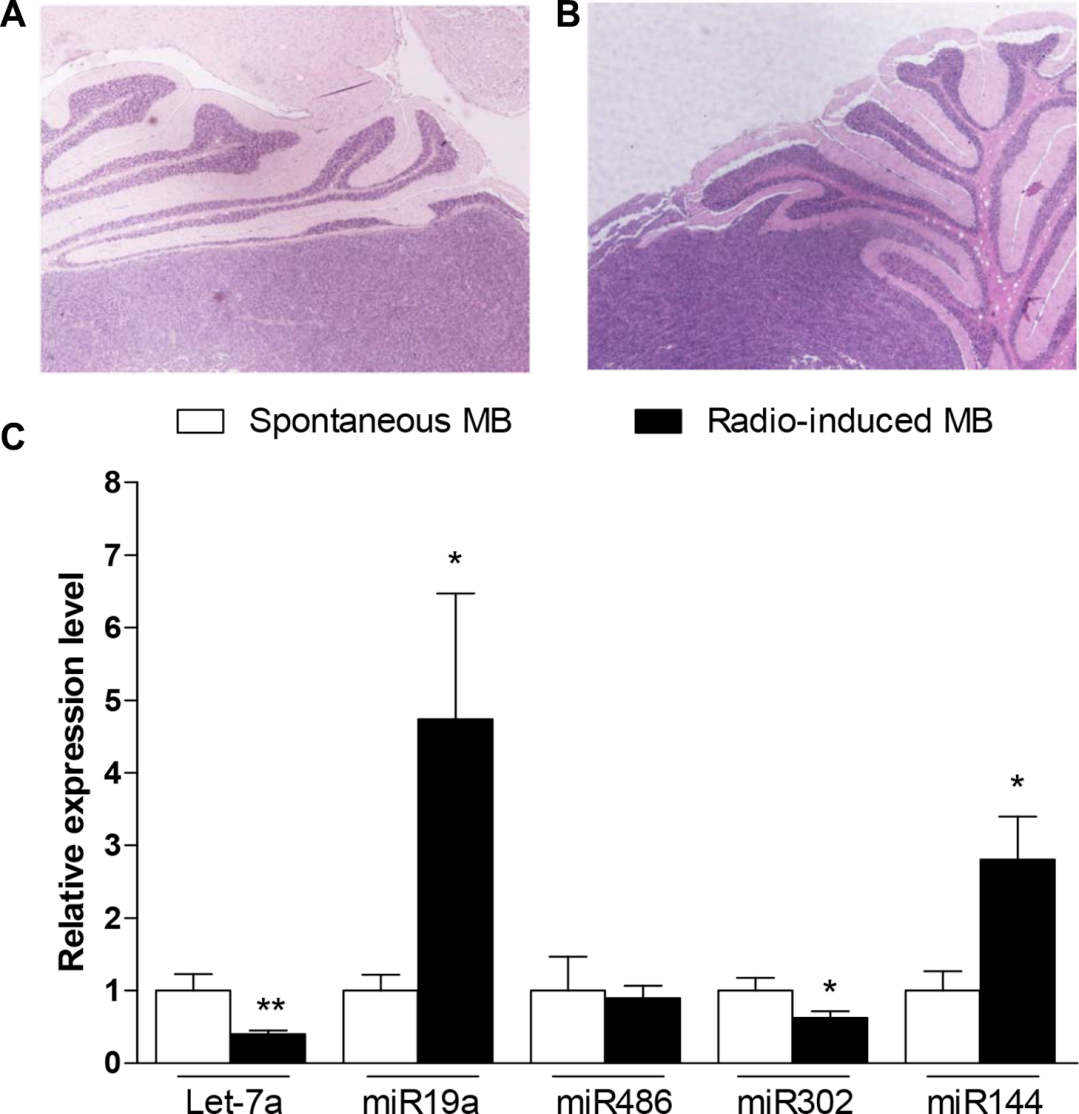
Real time PCR validation of selected statistically significant miRNAs from NGS and arrays on tumors Representative histological images of spontaneous (**A**) and radio-induced MB (**B**). (**C**) miRNAs expression in radio-induced MB with respect to the spontaneous one. Each data represent the mean and the standard deviation of three biological replicates. Student's *t-test* has been performed to evaluate the statistical significance of the comparisons. *P ≤ 0.05; **P ≤ 0.005.

To understand whether the miRNAs validated on GCPs (see Figure 7) could be responsible for MB development, we evaluated their expression level in spontaneous and radio-induced MBs (representative images in Figure 8A and 8B). As shown in Figure 8C, 4/5 of the analyzed miRNAs (i.e., let-7a, miR-19a, miR-144 and miR-302b) were significantly differentially expressed in spontaneous and radio-induced MB, according to their deregulation at short-term post-irradiation. The persistence of this modulation many weeks after exposure supports the hypothesis that these miRNAs and consequently the biological functions they control, may be involved in the extreme radiosensitivity of the *Ptch1*^+/−^ mouse model, determining a higher risk for radiation-induced cancer. Finally, miR-486 expression levels did not differ between spontaneous and radio-induced MB.

## DISCUSSION

For children and adolescents less than 20 year old, neoplasms of the CNS are the second most frequent childhood cancer; they account for more than 16% of all childhood malignancies. MB represents the most common pediatric brain tumor and comprises four distinct molecular variants. One of these is characterized by activation of the SHH pathway, driving approximately 25–30% of sporadic MB [19].

In the last two decades, many Shh signaling-based MB mouse models have been genetically engineered, providing an essential tool for uncovering the molecular and cellular basis of human diseases [20]. In particular, using mice with heterozygous deletion of exons 6 and 7 of *Ptch1* gene [21], we pioneered a protocol suitable to induce a high incidence (up to 80%) of MB after irradiation at P2 with 3 Gy of X-rays [13, 14]. The hypersensitivity to radiation is also a peculiar feature of the Gorlin syndrome, an autosomal dominant disorder due to a germline inactivation of the *PTCH1* gene, and individuals affected have a higher risk than the general population of developing tumors [22]. The link between deregulated Shh signaling and radiation-induced MB has not been sufficiently investigated. Within the past few years, however, the recognized role of miRNAs in almost all aspects of cell biology, among which regulation of the expression of components of cell pathways relevant to radiosensitivity, has gained huge attention [23].

In this study we developed an *ex vivo* experimental approach to shed light on miRNA repertoire revealed by NGS, and its integration into functional cellular networks, in WT and *Ptch1*^+/−^ GCPs, unirradiated or at short term after irradiation with a moderate dose of X-rays. By comparing results from NGS analysis, only a small number of miRNAs were in common in the two conditions. One of the miRNAs of interest is miR19a-5p, belonging to one of the best-known miRNA clusters, the miR-17∼92, which encodes six miRNAs (i.e., miR-17, miR-18a, miR-19a, miR-20a, miR-19b-1 and miR-92-1) [24]. The miR-17∼92 cluster is important in cell cycle, proliferation, apoptosis and other pivotal processes as well as in a wide array of diseases (i.e., in hematopoietic and solid cancers, and in immune, neurodegenerative and cardiovascular diseases) [25]. Importantly, it has been reported that Shh signaling pathway and miR-17∼92 collaborate during cerebellar development and in MB formation [26]; this functional interaction in GCPs is mediated in part by N-myc and C-Myc, both Shh effectors, which induce miR-17∼92 expression [27]. Furthermore, results from Murphy and colleagues [28], showed the therapeutic potential of 8-mer LNA-anti-miRs in inhibiting miR-17, 20a, 106b, and 93 (anti-miR-17) and miR-19a and 19b-1 (anti-miR-19) in two murine SHH-driven MB models. In absence of irradiation, here we show no difference in expression levels of three miRNAs belonging to miR-17∼92 cluster (miR-17, miR-19a, miR-20a; Figure 7), although at P2/3 GCPs are actively proliferating. Notably, at short term after irradiation, their expression in *Ptch1*^+/−^ GCPs was significantly higher compared with WT cells and a higher expression of the miR-19a characterizes radio-induced compared with spontaneous tumors. It has been shown that the miR-17∼92 cluster is positively upregulated by c-Myc thereby favoring tumorigenesis by enhancing cell proliferation and inhibiting apoptosis [29]. Very recent results provided evidence that ionizing radiation specifically induces c-MYC amplification; in fact, high-level c-MYC amplification has been found in primary mammary epithelial cells following radiation doses between 2 and 4 Gy and elevated c-MYC amplification was more common in human breast cancer which developed after radiotherapy compared with breast cancer without previous radiation exposure [30]. Supported by these considerations, we suggest that a functional loop between Shh deregulation and ionizing radiation exposure increases, *via* c/N-myc, the miR-17∼92 expression level immediately after irradiation, and this expression remains persistently upregulated in radio-induced MB compared with spontaneous tumors. This result could partly explain the increased radio-induced MB incidence, guided by a more proliferative status.

An important aspect highlighted by our analysis was the significant dowregulation of three different members of the let-7 family observed in unirradiated *Ptch1* mutant compared with WT cells. The let-7 family has gained notoriety owing to its regulation of stem cell differentiation, essential role in normal development, as well as its tumor suppressor function [31]. Regarding CNS, Wulczyn and colleagues [32] presented evidence for both transcriptional and post-transcriptional control mechanisms in the induction of let-7 family members during neural differentiation. Consistent with the identified pro-differentiation function of the let-7 family, our results underline that Shh-deregulated GCPs are characterized, at this early time of their postnatal development, by a lower differentiation level compared with WT cells. In a previous work, we showed that multipotent neural stem cells and fate-restricted progenitor cells strongly express stem cell markers, i.e. the transcription factor SRY (sex-determining region)-box 2 (Sox2), during embryonic development [33]. The level of Sox-2 expression remains high in GCPs also during post-natal cerebellum development [34], and has an effect on the proliferation rate of Shh-activated GCPs giving rise to a hyperproliferative progeny that is unable to exit the cell cycle, thereby driving Shh-associated MB development [35]. Importantly, it has been demonstrated that self-renewal and neuronal differentiation of neural precursors is controlled by Sox-2 that regulates the expression of LIN28, a suppressor of let-7 miRNA biogenesis [36]. These evidences explain the significant decreased expression of let-7 family we found in unirradiated *Ptch1*-deficient GCPs and suggest an involvement of these miRNAs in spontaneous MB development in *Ptch1*^+/−^ mice. Notably, although irradiation significantly decreases the expression level of all members of the let-7 family regardless of genotype, this effect is particularly marked in *Ptch1* mutant cells suggesting cooperation of radiation insult with Shh deregulation *en route* to stem cell deregulation and tumorigenesis. The correlation between let-7 family and the immediate cellular response to irradiation was described by Weidhaas and colleagues in normal and cancer cell lines [37]; they found down-regulation of most members of the let-7 family within 2h after irradiation in both cancerous and normal lung epithelium cells. A radiation-induced decrease of let-7a and let-7b expression was also observed in radiation-sensitive tissues *in vivo* and it was shown to correlate with altered expression of p53 protein, a key mediator of DNA damage response cascade following exposure to ionizing radiation [38, 39]. Our data obtained by IPA's MAP clearly show a key role of let-7a in the DNA damage repair activity (see Figure 6), corroborating the hypothesis that a different DNA repair machinery characterizes the DNA damage response induced by radiation in GCPs in association to *Ptch1* deficiency, as evidenced by our pathway analysis of NGS and mRNA arrays (Figures 3 and 4).

Another actor involved in the more undifferentiated status of *Ptch1*^+/−^ cells is miR-302b. It represses premature expansion of progenitors and production of post-mitotic neurons functioning opposite to let-7, as its loss leads to acceleration, rather than delay in differentiation [40]. Even though not statistically significant, we found a 1.3-fold decreased expression of miR-302b in unirradiated *Ptch1*-mutant compared with WT cells; after irradiation, this difference became statistically different (1.6-fold decrease). As miR-302b is involved in radio-resistance via directly targeting AKT1 and RAD52, two critical regulators of the cellular response to radiation [41], its lower level in *Ptch1*^+/−^ GCPs could be partly responsible for the radio-resistance of these cells.

miRNome analysis identified other interesting and significantly altered miRNAs, among which miR-144. In agreement with previous work showing miR-144 upregulation by aberrant Shh activation [42], we found a significantly higher level of miR-144 in unirradiated *Ptch1*^+/−^ GCPs than in WT cells (2-fold increase). After irradiation, its expression increased significantly in WT cells (1.48-fold increase), and was strongly upregulated in *Ptch1*^+/−^ GCPs (2.5-fold increase); moreover, radio-induced MBs express significantly more miR-144 than spontaneous MBs. These observations suggest a synergism between irradiation and Shh pathway activation in the control of miR-144 expression level. Altered expression of miR144 causes post-transcriptional repression of the Nuclear factor E2-related factor 2 (Nrf2), a transcription factor that upregulates expression of a battery of antioxidative genes which constitute the cellular response to oxidative stress and xenobiotic damage [43]. Thus, it is intriguing to speculate that an impairment of the cellular redox status and adaptive cellular response to oxidative stress in *Ptch1*^+/−^ GCPs, caused by miR-144 overexpression, contributes to the higher incidence of radio-induced tumors in this mouse model.

Finally, *Ptch1*^+/−^ GCPs express higher levels of miR-486 than WT cells, irrespective of irradiation. However, after irradiation, mir-486 expression level in *Ptch1*^+/−^ GCPs was significantly increased compared with unirradiated cells. miRNA enrichment and pathway analysis revealed that, in the cell compartment analyzed, miR-486 directly targets components of insulin growth factor (IGF) signaling. This result is consistent with our previous data showing that IGF-I overexpression in GCPs fosters external granular layer proliferative lesions through a mechanism favoring proliferation over terminal differentiation, and acting as a landscape for tumor growth [44]. In addition, it has been reported that miR-486 is regulated by p53 demonstrating a key role of this miRNA in controlling G1/S transition following DNA damage [45]. Due to the regulation of miR-486 by p53 and its role in cell-cycle control, it is conceivable that miR-486 acts only at short term after irradiation, because both p53 activation and inhibition of cell growth decrease out just 24 hours after irradiation. According with this assumption, evaluation of miR-486 expression in tumors shows the same level in spontaneous and radio-induced MB.

In summary, we have developed an *ex vivo* experimental approach to shed light on miRNA repertoire revealed by NGS, and its integration into functional cellular networks, in unirradiated WT and *Ptch1*^+/−^ GCPs and at short term after irradiation with 1 Gy of X-rays. We identified a subset of miRNAs, controlling different biological functions, whose expression was altered in GCPs by radiation alone or in combination with Shh-deregulation. In addition, our results support the model of cancer as an alteration of normal development - in these experiments caused by Shh deregulation alone or in combination with radiation damage - as many miRNAs were similarly expressed in primary cultures of GCPs and in MB. In combination with bioinformatics analysis, the data obtained have a number of potential implications for the further development of radiation risk research based on knowledge of miRNA mechanisms, especially cancer risks associated with occupational, diagnostic and environmental exposures which typically occur at low radiation doses.

## MATERIALS AND METHODS

### Cell isolation and culture conditions

Pups were genotyped at P1, as described [21]. At P2, GCPs were purified from mouse cerebella as *per* Hatten and colleagues [46], with minor modifications. Briefly, cerebella from WT and *Ptch1*^+/−^ mice were dissected away from the remaining brain. The *pia mater* was removed, and cerebella were treated with papain and dissociated into a single-cell suspension. Cells were separated on a density step gradient of 35% and 60% Percoll Plus solution (GE Healthcare Life Sciences, Uppsala, Sweden). GCPs have the highest density of all cell types in cerebellum and can be separated from a less dense fraction that contains glial cells, Purkinje cells, and large interneurons. Purified GCPs were further enriched by panning on tissue culture dishes to remove adherent fibroblasts. Non-adherent cells were plated at a density of 1 × 10^6^ cells/ml in dishes precoated with a poly D-lysine solution (Sigma-Aldrich, St. Louis, MO) with 3 μg/ml Shh (Peprotech, Rocky Hill, NJ). Cells were grown in Neurobasal medium with B27 without vitamin A.

### Irradiation

The day after purification, cultured GCPs were exposed to a single dose of 1 Gy of X-rays or left untreated. Irradiation was performed using a Gilardoni CHF 320 G X-ray generator (Gilardoni S.p.A., Mandello del Lario, Italy) operated at 250 kVp, with HVL = 1.6 mm Cu (additional filtration of 2.0 mm Al and 0.5 mm Cu).

### RNA isolation, library preparation and next generation sequencing (NGS)

Four hours after irradiation, cultured GCPs were collected and total RNA was extracted using miRNeasy kit (≠ 217004; QIAGEN, Milan, Italy) according to the manufacturer's instructions. Total RNA was (1 μg) converted into miRNA NGS libraries using NEBNEXT library generation kit (New England Biolabs Inc., Beverly, MA) following manufacturer's instructions. Samples were sequenced on the Illumina NextSeq 500 System.

### Sequencing data analysis for differential expression gene and pathway enrichment

All sequencing data analysis was performed using the R platform (http://www.r-project.org/) and the open-source Bioconductor libraries. Data were filtered based on sequence counts (i.e. >1 reads *per* million in at least 2 samples) and pairwise comparisons of differential miRNA expression were performed using edgeR package [47–49]. Statistically significant miRNAs (*P* < 0.05) were used for gene/miRNA enrichment analysis with Cytoscape plug-in “ClueGo” (v.2.1.7) and “CluePedia” (v.1.1.7) [50]. For each miRNA list, enrichment was performed for individual miRNAs employing the miRanda database (miRanda score threshold = 0.6) and showing the top 20 predicted target genes corresponding to each miRNA. Subsequently, predicted target genes and miRNAs were selected to find the affected functions on the Reactome database [51–52].

### Array analyses

Total RNAs (250 ng) were reverse-transcribed with the RT² First Strand Kit (≠ 330401, QIAGEN) for first strand cDNA synthesis and were used to probe, according to the manufacturer's instructions, following QUIAGEN arrays: DNA Repair PCR Array (≠ PAMM-42Z) and Mouse DNA Damage Signaling Pathway (≠ PAMM-029Z). qPCR was carried out by StepOnePlus™ Real-Time PCR System (Applied Biosystems, Life Technologies, Monza, Italy).

Total RNAs (250 ng) were reverse-transcribed with miScript II RT Kit (≠ 218160, QIAGEN) for cDNA synthesis of miRNAs and were used, according to the manufacturer's instructions, to probe the following miRNA PCR QIAGEN arrays: Mouse Brain Cancer miRNA PCR Array (≠ MIMM-108Z) and Mouse miFinder miRNA PCR Array (≠ MIMM-001Z). Both arrays contained 96 lyophilized mouse miRNA sequences along with endogenous controls. miRNA array analysis was performed using two biological replicates and data analysis was performed with PCR Array Data Analysis Software (QIAGEN).

Network analysis was performed by Cytoscape plug-in “ClueGo” (v.2.1.7), using the KEGG database, for significantly up- and down- deregulated mRNAs (*P* < 0.05). Gene nodes color code (red = up-regulated, green = down-regulated) is kept for the identified enriched KEGG pathways, where the color gradient reflects the proportion of up/down regulated genes associated to the pathways (Figure 4).

miRNAs (with |FC|>1.1) were analyzed with the software tool Ingenuity Pathway Analysis (IPA) (INGENUITY System, http://www.INGENUITY.com) [53] and a pathway enrichment analysis of the miRNA dataset was performed. IPA's “Disease & Function” tool was queried to select specific functional endpoints and focus on both key known and predicted target molecules. The use of IPA's Molecule Activity Predictor (MAP) allowed simulating the effects of measured mRNA and miRNAs on the selected endpoints.

### miRNAs validation by qRT-PCR

Primers QIAGEN used for validation were: Mm_miR-302b (≠ MS00002058); Mm_miR-486 Primer Assay (≠ MS00029246); Mm_miR-144 (≠ MS00032326); Hs_SNORD61 (≠ MS00033705); Mm_miR-19a (≠ MS000011403) Other miRNAs analyzed by TaqMan MicroRNA Assay Kits (ThermoFisher Scientific, Milan, Italy) were: mmu-miR-17-5p (Assay Name 002308); mmu-miR-20a-5p (Assay Name 000580); mmu -let-7a (Assay Name 000377), mmu-let-7b-5p (Assay Name 002619); mmu-miR-let-7c-5p (Assay name 0000379); U6 snRNA (Assay Name 01973). In real-time qPCR experiments, each sample was run in triplicate in three independent experiments. To determine if the dose and the genotype determine a statistically significant differential response, two-way ANOVA with multiple comparisons (with Bonferroni post-hoc tests to compare replicate means) were performed. Where not otherwise stated, statistical significance (*P*) was calculated by two-tailed Student's *t-test*.

## SUPPLEMENTARY MATERIALS FIGURES


