# Supplementary material for: Ex vivo miRNome analysis in Ptch1+/− cerebellum granule cells reveals a subset of miRNAs involved in radiation-induced medulloblastoma
Source: Oncotarget. 2016 Sep 10;7(42):68253–69. doi: 10.18632/oncotarget.11938 (PMC5356552; doi:10.18632/oncotarget.11938)
Supplement: Supplementary file 1 [file oncotarget-07-68253-s001.pdf]

Ex vivo miRNome analysis in *Ptch1*$^{+/-}$ cerebellum granule cells reveals a subset of miRNAs involved in radiation-induced medulloblastoma

Supplementary Materials

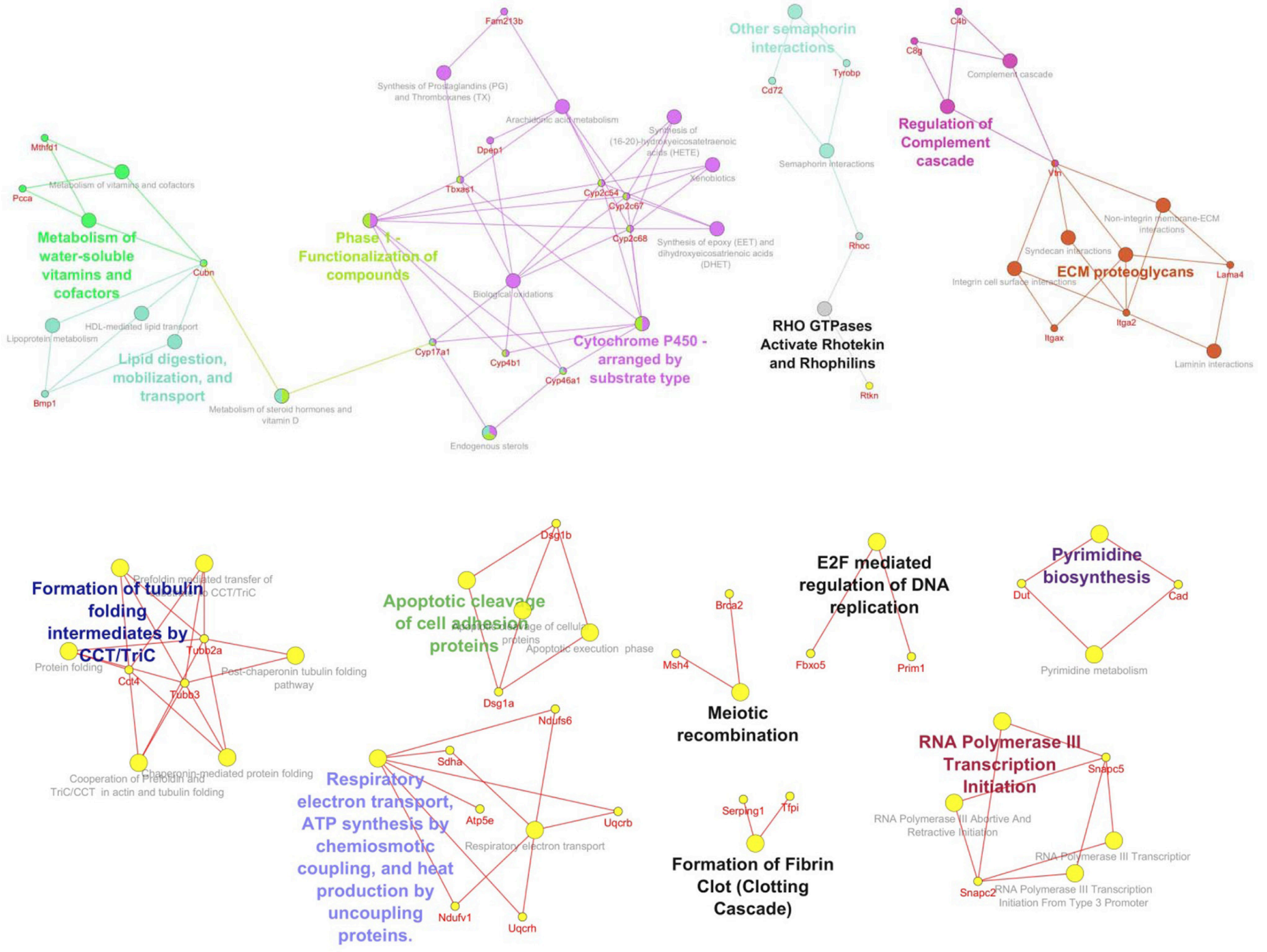

Supplementary Figure S1: Pathway enrichment analysis was performed on the statistically significant miRNAs altered in unirradiated *Ptch1*$^{+/-}$ GCPs versus WT GCPs. Focus on the other Reactome-based pathways and mRNAs, not shown in Figure 2A, predicted to be altered by the differentially expressed miRNAs.
